# Brassinosteroids modulate ABA-induced stomatal closure in Arabidopsis

**DOI:** 10.1093/jxb/erw385

**Published:** 2016-10-17

**Authors:** Yunmi Ha, Yun Shang, Kyoung Hee Nam

**Affiliations:** Department of Biological Sciences, Sookmyung Women’s University, Seoul 04310, Republic of Korea

**Keywords:** ABA sensitivity, ABA-induced stomatal closure, abscisic acid (ABA), brassinosteroid (BR), ROS production, stomatal movement.

## Abstract

Stomatal movement in response to water availability is an important physiological process in the survival of land plants. The plant hormone abscisic acid (ABA) and brassinosteroids (BRs) regulate stomatal closure. The physiological functions of ABA and BRs, including germination, cell elongation and stomatal movement, are generally known to be antagonistic. Here, we investigated how BRs affect stomatal movement alone and in combination with ABA. We demonstrate that brassinoslide (BL), the most active BR, promotes stomatal closure in an ABA-independent manner. Interestingly, BL also inhibited ABA-induced stomatal closure when a high concentration of BL was added to ABA. Furthermore, we found that the induction of some genes for reactive oxygen species (ROS) generation by ABA (*AtrbohD*, *NIA1* and *NIA2*) and subsequent ROS production were repressed by BL treatment. The BR signaling mutant *bri1-301* failed to inhibit ABA-induced stomatal closure upon BL treatment. However, *BRI1*-overexpressing transgenic plants were hypersensitive to ABA during stomatal closure, and BL reversed ABA-induced stomatal closure more completely than in wild type plants. Taken together, these results suggest that BRs can positively and negatively modulate ABA-induced stomatal closure. Therefore, interactions between ABA and BR signaling are important for the regulation of stomatal closure.

## Highlight

Brassinosteroids alone promote stomatal closure, and in combination with ABA, they positively and negatively modulate ABA-induced stomatal closure in Arabidopsis.

## Introduction

Stomatal closure under water-deficient conditions is a cellular process common to most land plants. The proper development of stomata and regulation of stomatal movements are critical for the regulation of water levels and for facilitating the productivity of plants. Stomatal movement is regulated not only by various environmental conditions, such as light, CO_2_ (Hubbard *et al.*, 2012), nitric oxide (NO) (Sugiyama *et al.*, 2012), and ozone (Vahisalu *et al.*, 2010), but also by multiple plant hormones, such as abscisic acid (ABA), ethylene, methyl jasmonate, and brassinosteroids (BRs) (Suhita *et al.*, 2004; Desikan *et al.*, 2006; Haubrick *et al.*, 2006; An *et al.*, 2008).

ABA is a major plant hormone that is increased under a variety of abiotic stresses (Chinnusamy *et al.*, 2004; Nakashima *et al.*, 2009). It is detected by the soluble receptor PYRABACTIN RESISTANCE1 (PYR1)/PYR1-LIKE (PYL)/REGULATORY COMPONENT OF ABA RECEPTORS (RCAR) (Park *et al.*, 2009; Klingler *et al.*, 2010). This inhibits ABA-insensitive 1 (ABI1), a clade A-type protein phosphatase 2C (PP2C) (Vlad *et al.*, 2009), which releases major positive regulators for ABA signaling, including the cytoplasmic protein kinases, sucrose non-fermenting 1-related subfamily 2 (SnRK2.6)/OPEN STOMATA1 (OST1), SnRK2.2 and SnRK2.3, from inhibition by PP2Cs (Ng *et al.*, 2011). Free activated OST1 activates the S-type and R-type anion channels SLAC1 and QUAC1, respectively (Lee *et al.*, 2009; Vahisalu *et al.*, 2010; Imes *et al.*, 2013), and the K^+^ channel KAT1 (Mori *et al.*, 2000; Sato *et al.*, 2009) in the plasma membrane of guard cells, resulting in stomatal closure. OST1 also activates AtrbohF, a subunit of NADPH oxidase, resulting in the production of reactive oxygen species (ROS) (Sirichandra *et al.*, 2009). Further downstream signaling through the activation of OST1 activates the transcription factors ABI3, ABI4, and ABI5 (Feng *et al.*, 2014), leading to changes in the expression of genes involved in the regulation of seed germination and root and hypocotyl growth (Finkelstein *et al.*, 2002; Nakashima *et al.*, 2009; Hayashi *et al.*, 2014).

The cellular physiological functions of ABA are thought to be antagonistic to those of BRs. In seed maturation and germination, embryonic ABA maintained seed dormancy, preventing precocious germination (Seo *et al.*, 2009) and exogenous ABA delayed seed germination, while several ABA-deficient mutants, such as *nced6*, *nced9*, *aba2* and *aao3*, showed a higher percentage of germination than wild type plants (Seo *et al.*, 2006; Preston *et al.*, 2009). In comparison, 24-epibrassinolide (EBR) treatment rescued germination of gibberellic acid (GA)-deficient and GA response mutants (Steber *et al.*, 1998; Steber and McCourt, 2001; Ullah *et al.*, 2002). Furthermore, germination of the BR-deficient mutant *det2* and the BR signaling mutant *bri1-1* was more delayed in response to ABA than it was in wild type plants (Steber and McCourt, 2001), indicating that BRs promote seed germination. Hypocotyl and root elongation were more severely inhibited in the *det2* and *bri1-9* mutants in response to ABA than in the wild type (Xue *et al.*, 2009).

However, the functional relation of BRs with ABA in stomatal closure, which is an ABA-induced phenotype, seems to be more complex. On one hand, the BR-deficient mutants *sax1* and *det2* and BR signaling mutant *bri1-9* showed enhanced ABA-induced stomatal closure (Ephritikhine *et al.*, 1999; Xue *et al.*, 2009), supporting the hypothesis that ABA sensitivity is inversely related to BR level, as suggested previously (Steber and McCourt, 2001). On the other hand, a BR and ABA cooperated to reduce stomatal transpiration in *Vicia faba* guard cells in response to drought (Haubrick *et al.*, 2006). In maize leaves, BR-induced NO production and subsequent NO-activated ABA biosynthesis were reported to exert water stress tolerance (Zhang *et al.*, 2011). Recently, EBR was reported to induce stomatal closure in Arabidopsis leaves (Shi *et al.*, 2015). Taken together, these findings support the hypothesis that BRs promote stomatal closure. Moreover, Xia *et al* (2014) reported a dose-dependent effect of EBR on stomatal closure and opening in tomato leaves (Xia *et al.*, 2014). A low EBR concentration (<0.1 μM) induced stomatal opening, whereas stomatal closure was observed under higher concentrations of EBR.

BR signaling is triggered by BR binding to BRI1, a leucine rich-repeat (LRR) serine/threonine receptor-like kinase (RLK) located in the plasma membrane (Li and Chory, 1997; Wang *et al.*, 2005). BR-bound BRI1 recruits another type of LRR-RLK, BAK1, to form a functional receptor complex (Li *et al.*, 2002; Nam and Li, 2002). Recently, we reported the reduced ABA sensitivity of *bak1* in stomatal closure and showed that BAK1 interacts with OST1 near the plasma membrane. Moreover, brassinoslide (BL) negatively affected the ability of BAK1 to form a complex with OST1 in the presence of ABA (Shang *et al.*, 2016). Taken together, these results make it difficult to assess the function of BRs in the regulation of stomatal movement.

We have noted that, thus far, many experiments performed to measure stomatal apertures have involved single applications of ABA or BR to BR-related or ABA-related mutants, respectively. In this study, we further investigated how BR affects stomatal movement on its own or in combination with ABA. BR promotes stomatal closing in Arabidopsis, as does ABA. However, in combination with ABA, BR concentrations exceeding a threshold level antagonized the effects of ABA on stomatal closure. Co-treatment of BR and ABA repressed the expression of genes responsible for ABA-induced ROS production, resulting in decreased ROS signaling. Using BR-deficient and signaling mutants, and a BR-hypersensitive BRI1-overexpressing transgenic plant, we further investigated how BR affects the movement of stomata following closure in response to ABA treatment. We found that BR signaling is required for the plants to maintain ABA sensitivity and to inhibit ABA-induced stomatal closure by BR. Taken together, these results suggest that interactions between ABA and BR signaling are important for the regulation of stomatal closure.

## Materials and methods

### Plant material and growth conditions

*Arabidopsis thaliana* Columbia-0 (Col-0) was used as the wild type plant in all experiments, except when *aba2-1* [Landsberg (L*er*) background] and *dwf4-1* [Wassilewskija-2 (Ws-2) background] were examined. Other mutants and the *BRI1-GFP* transgenic plants used in this study were from our own lab stocks. Brassinazole (BRZ) was provided by Prof. Seong-Ki Kim from Chung-Ang University. Seeds were sterilized with 75% ethanol containing 0.05% Tween-20 for 15 min, washed twice in 95% ethanol and once in 100% ethanol, and then placed onto 1/2 Murashige and Skoog (MS) (Duchefa Biochemie) plates containing 0.8% phytoagar. All plants were grown at 22 °C under long-light conditions (16 h light–8 h dark).

### Stomatal aperture measurement

We followed the methods described by Shang *et al.* (2016) to measure stomatal apertures. Abaxial surfaces of cotyledons from 10-d-old seedlings were incubated in stomatal opening solution (50 mM KCl, 10 μM CaCl_2_, 10 mM MES, pH 6.15) in a growth chamber with light intensity of 130 μmol m^–2^ s^–1^ at 22 °C. Various reagents, such as ABA (Sigma-Aldrich), BL (Sigma-Aldrich), BRZ, H_2_O_2_ and sodium nitroprusside (SNP) (Sigma-Aldrich) were added to the opening solution for the indicated period. Stomatal opening was evaluated by measuring the width and length of the stomata observed under a microscope (Leica, DM2500) and was calculated by determining the width/length ratio.

### Quantitative RT-PCR

To analyse the quantitative expression of genes involved in H_2_O_2_ and NO production in response to ABA or BL, RNA was isolated from 10-d-old seedlings using the TRIzol reagent (Sigma-Aldrich) after the seedlings were treated with 1 μM ABA or BL, or together for 1 h. First-strand cDNA was synthesized using M-MLV reverse transcriptase (Promega) and the oligo(dT) 15 primer using 1 μg RNA. The same aliquot of first-strand cDNA was used as a template in the second PCR with gene-specific primers. Quantitative RT-PCR was performed with *UBQ*5 as an internal control and analysed with the Step-one Plus Real Time PCR system using the same cDNA and SYBR Green PCR Master Mix as described previously (Applied Biosystems, Foster City, CA, USA). Data were normalized to *UBQ*5 expression. Primer sequences used in this experiment are provided in Supplementary Table S1 at *JXB* online.

### Detection of H_2_O_2_ and NO production

Abaxial leaf epidermal peels from 4-week-old plants were used to determine H_2_O_2_ and NO production. Epidermis was incubated in the stomatal opening solution containing ABA or BL alone, or in combination, and were then incubated in 100 µM 2′,7′-dichlorodihydrofluorescein diacetate (H_2_DCF-DA) (Fisher Scientific) for 15 min to detect H_2_O_2_ production. To detect NO production, epidermis was incubated with 200 µM 4-amino-5-methylamino-2′,7′-difluorofluorescein diacetate (DAF-FMDA) (Thermo Fisher Scientific) for 20 min. Dyes were washed off with distilled water. H_2_O_2_ and NO production in guard cells was observed under a fluorescence microscope (Leica, DM2500 with a fluorescence module; Fluo Illuminator L4/23) and the L5 filter system (excitation BP480/40, emission BP527/30). To prevent photo-oxidation of H_2_DCF-DA, all fluorescence images were collected with a single rapid capture (150.8 ms frame^–1^) at ×400 magnification, as described by Shang *et al.* (2016).

## Results

### Treatment with ABA or BR induces stomatal closure

**Fig. 1.**
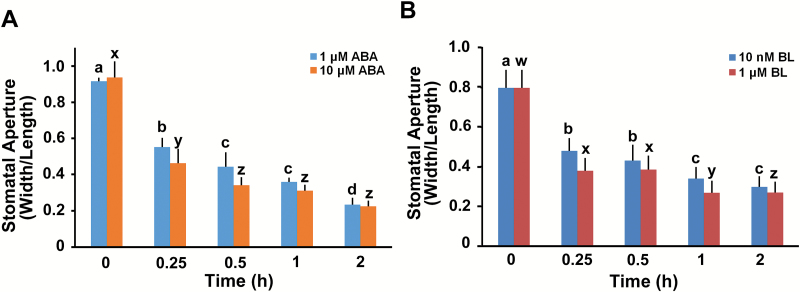
Abscisic acid (ABA) and brassinolide (BL) induced stomatal closure. ABA (1 or 10 μM; A) or BL (10 nM or 1 μM; B) was added to the opening solution and the seedlings were incubated for the indicated times. Stomatal closure was measured as the width/length ratio of the cotyledon stomata. Experiments were independently repeated three times (*n*=30 each time). Error bars indicate standard errors. Values labeled with different letters are statistically different analysed by one-way ANOVA (*P*<0.05). (This figure is available in color at *JXB* online.)

To determine the roles of BRs and ABA in stomatal closure, we examined ABA- or BR-induced stomatal movements for various time periods. Stomatal closure in cotyledons of 10-d-old seedlings was detected 15 min after ABA treatment. Two-hour ABA treatment almost completely closed the stomata of the cotyledons (Fig. 1A). Treatment with brassinolide (BL), which is the most bioactive BR, also induced stomatal closure over the duration of treatment similarly to ABA (Fig. 1B). We observed that stomata were closed proportionally to the concentration of BL when treated for a short period (15 min). However, the degree of stomatal closure by BL showed little difference at concentrations higher than 1 nM for longer period (2 h) (Supplementary Fig. S1). These results are consistent with reports that BL causes stomatal closure (Haubrick *et al.*, 2006; Shi *et al.*, 2015), as observed in response to ABA. Furthermore, BR-induced stomatal closure is consistent with results reported by Xia *et al.* (2014), in which high concentrations of EBR (more than 1 μM) induced stomatal closure of tomato in the light (Xia *et al.*, 2014).

**Fig. 2.**
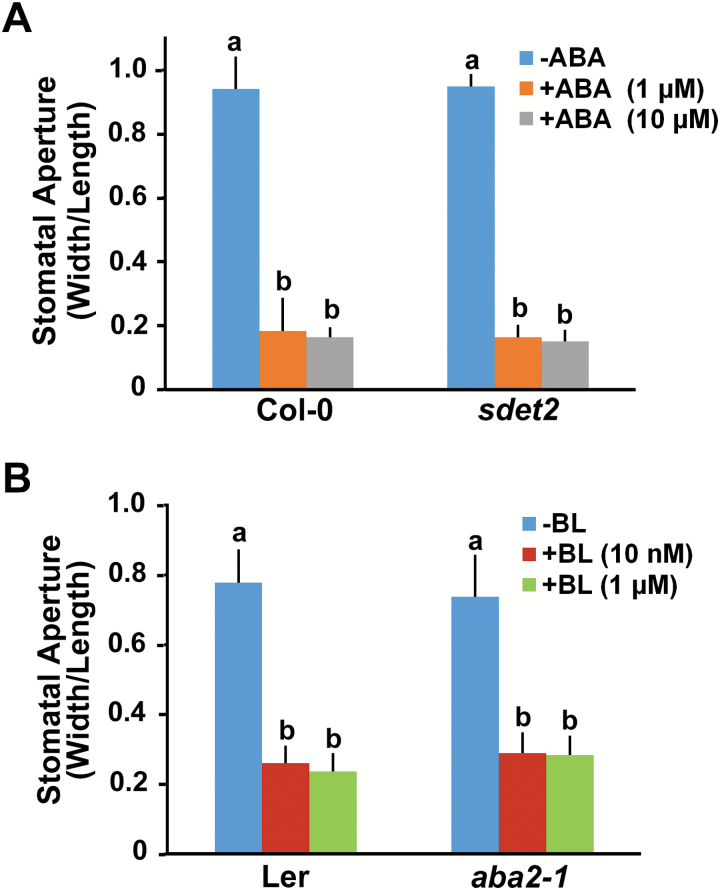
BR- or ABA-deficient mutants showed normal sensitivity to ABA or BL, respectively, in stomatal closure. ABA-induced stomatal closure in the *sdet2* mutant (A) and BL-induced stomatal closure in the *aba2-1* mutant (B) were measured after 2 h incubation with each hormone and compared with the corresponding wild type plants. ABA or BL with indicated concentrations was added to the opening solution and the seedlings were incubated for an additional 2 h. The stomatal aperture was determined as the width/length ratio of the cotyledon stomata. Experiments were independently repeated twice (*n*=30 each time). Error bars indicate standard errors. Values labeled with different letters are statistically different analysed by two-way ANOVA (*P*<0.05). (This figure is available in color at *JXB* online.)

To further examine whether stomatal closure in response to BL or ABA is independent of each hormone, we measured the stomatal aperture from *sdet2* (Park *et al.*, 2014), a BR biosynthetic mutant, and *aba2-1*, an ABA biosynthetic mutant (Schwartz *et al.*, 1997) under ABA or BL treatment, respectively. In this study, we used the *sdet2* mutant instead of the original *det2* mutant, because *sdet2* was newly segregated from the unknown additional mutation that led to severe inhibition of root elongation in *det2* plants (Park *et al.*, 2014). The *sdet2* mutant still carries the mutation in the *DET2* gene, resulting in a rounded and dark-green aerial rosette and dwarfed stature. We also confirmed that treatment with BL partially rescued *sdet2* shoot phenotypes (Supplementary Fig. S2). Then, we determined stomatal aperture of *sdet2* and *aba2-1* in response to low and high concentrations of ABA and BL, respectively. The *sdet2* mutant remained sensitive to ABA-induced stomatal closure under both low (1 μM) and high (10 μM) concentrations of ABA treatment (Fig. 2A). It showed the same sensitivity to ABA even at concentrations lower than 1 μM compared with wild type plant (Supplementary Fig. S3). Stomata of the *aba2-1* plants were also closed in response to both low (10 nM) and high (1 μM) and concentrations of BL treatment (Fig. 2B). Another BR-biosynthetic mutant, *dwf4-1*, and ABA biosynthetic mutant, *aao3-4*, showed similar trends in stomatal closure in response to BL and ABA, respectively (Supplementary Fig. S4A, B). In addition, the ABA signaling mutant *abi1-1* (Wu *et al.*, 2003) showed similar stomatal aperture closing to that of wild type plants in response to BL, while ABA-induced stomatal closure was suppressed (Supplementary Fig. S4C). These results suggested that BL and ABA can cause stomatal closure independently.

### ABA-induced stomatal closure was reversed by a high concentration of BR

**Fig. 3.**
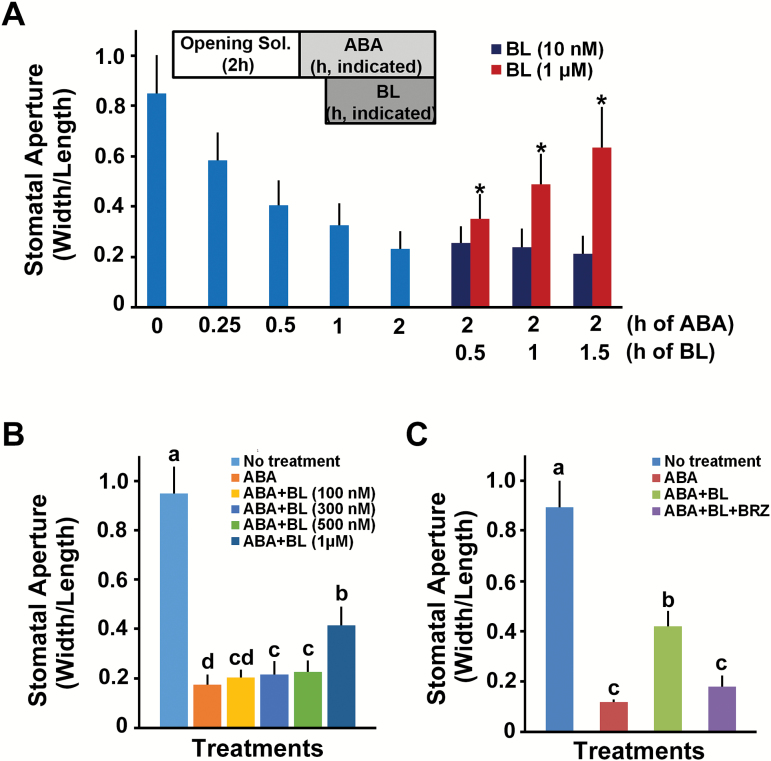
High concentrations of BL inhibited the ABA-induced stomatal closure. (A) ABA-induced stomatal closure was measured with or without BL treatment for indicated times. Low (10 nM) or high (1 μM) concentrations of BL were added to the ABA-containing solution for the indicated times. Experiments were independently repeated three times (*n*=50 each time). Error bars indicate standard errors (*P<0.0001, compared with the corresponding samples treated with ABA only). (B) Effects of BL concentrations on the inhibition of ABA-induced stomatal closure. Different concentrations of BL were added to the ABA-containing solution for 1.5 h. Experiments were independently repeated twice (*n*=30 each time). (C) Effect of BRZ on BL inhibition of ABA-induced stomatal closure. BRZ (1 μM) was applied 30 min after BL (1 μM) to the ABA-containing solution. Experiments were independently repeated twice (*n*=30 each time). In all experiments, 1 μM ABA was used. Error bars indicate standard errors. In (B) and (C), values labeled with different letters are statistically different analysed by one-way ANOVA (*P*<0.05). (This figure is available in color at *JXB* online.)

As ABA is a major hormone that affects stomatal movement in various plants (Du *et al.*, 2014; Qu *et al.*, 2014; Tombesi *et al.*, 2015), and both BL and ABA induced stomatal closure independently as shown in Fig. 1, we questioned whether BL acts additively on ABA-induced stomatal closure. Therefore, we examined stomatal closure induced by ABA following the addition of BL for various time periods. Stomata were almost completely closed in 2 h in the presence of 1 μM ABA alone. Addition of 10 nM of BL for different time periods did not affect ABA-induced stomatal closure (Fig. 3A). However, surprisingly, we observed that ABA-induced stomatal closure was reversed proportional to the BL exposure time when a high concentration of BL (1 μM) was added to ABA (Fig. 3A). This reversal of ABA-induced stomatal closure by a high concentration of BL was also observed when the higher concentration of ABA (10 μM) was used (Supplementary Fig. S5). To further examine the effects of BL concentration on ABA-induced stomatal closure, we monitored the effect of increasing concentrations of BL, and the effects of BRZ, a biosynthetic inhibitor of BRs (Asami *et al.*, 2000). We observed that ABA-induced stomatal closure was inhibited by BL in a dose-dependent manner. At concentrations higher than 500 nM, BL treatment opened the stomata that had closed in response to ABA (Fig. 3B). To reverse this pattern, co-treatment of BRZ with BL partially recovered ABA sensitivity in stomatal movement when compared with the effect of the BL treatment alone (Fig. 3C). Taken together, these results suggest that exogenously applied BL antagonizes ABA-induced stomatal movements dose-dependently.

### BR negatively affects ABA-induced H_2_O_2_ and NO production

**Fig. 4.**
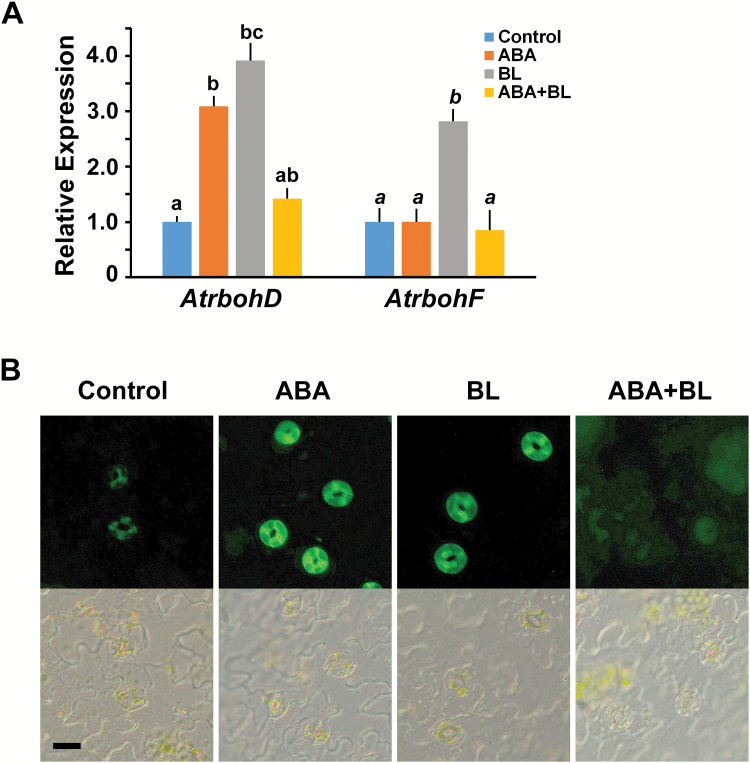
High concentrations of BL inhibited ABA-induced ROS production. (A) Relative expression of *AtrbohD* and *AtrbohF* in response to ABA and BL alone, and as a co-treatment. qRT-PCR analyses were performed in triplicate using RNA isolated from 10-d-old wild type Col-0 seedlings. Data were normalized to the expression of *ubiquitin*. Experiments were independently repeated twice. Error bars indicate standard error. Values labeled with different letters (roman and italic, respectively) are statistically different analysed by one-way ANOVA (*P*<0.05). (B) ROS production in response to each condition was detected by fluorescent H_2_DCF-DA in the guard cells (upper panels). Lower panels show the bright field images corresponding to the same region of the epidermal tissues. Scale bar indicates 20 μm.

**Fig. 5.**
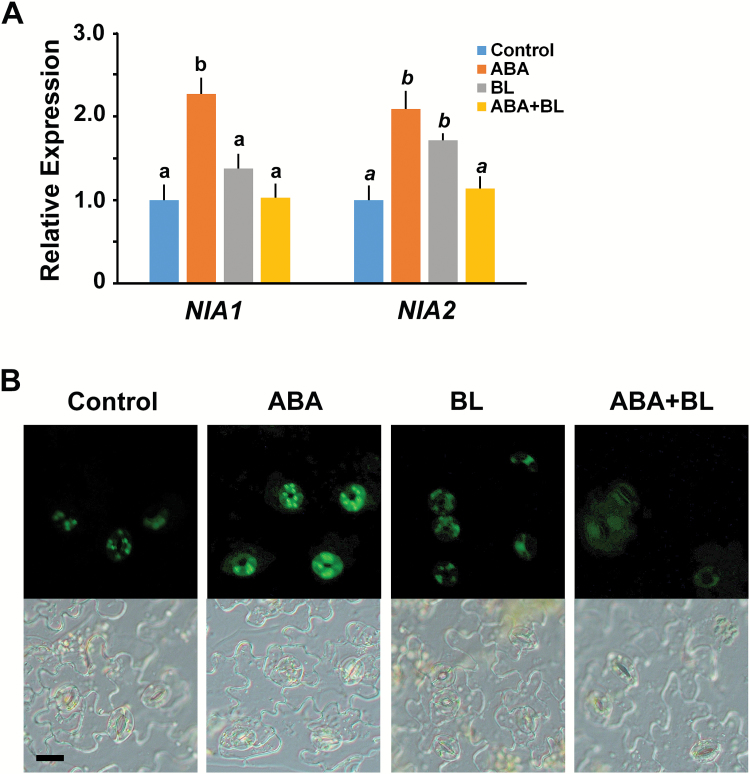
High concentrations of BL inhibited ABA-induced NO production. (A) Relative expression of *NIA1* and *NIA2* in response to ABA and BL alone, and in combination. qRT-PCR analyses were performed in triplicate using RNA isolated from 10-d-old wild type Col-0 seedlings. Data were normalized to the expression of *ubiquitin*. Experiments were independently repeated twice. Error bars indicate standard error. Values labeled with different letters (roman and italic, respectively) are statistically different analysed by one-way ANOVA (*P*<0.05). (B) NO production in response to each condition was detected by fluorescent DAF in the guard cells (upper panels). Lower panels show the bright field images corresponding to the same region of the epidermal tissues. Scale bar indicates 20 μm.

ROS, H_2_O_2_, and NO are signaling intermediates in stomatal closure in response to plant hormones as well as environmental stimuli (Pei *et al.*, 2000; Desikan *et al.*, 2002; Kolla *et al.*, 2007). ABA-induced ROS or NO production is a pre-requisite for stomatal closure (Kolla *et al.*, 2007; Gayatri *et al.*, 2013). Therefore, we wanted to determine whether ROS production was affected following co-treatment with ABA and BL, leading to the BL-induced inhibition of ABA on stomatal closure. First we examined the expression of genes encoding the proteins required for H_2_O_2_ and NO biosynthesis in wild type plants. Expressions of *AtrbohD* and *AtrbohF*, which encode NADPH oxidase catalytic subunits required for ROS production, were induced by ABA in guard cells (Kwak *et al.*, 2003). Consistent with a previous report, the expression of *AtrbohD* was increased around three-fold by ABA. However, the expression of *AtrbohF* was not changed by ABA. We also found that the expression of *AtrbohD* and *AtrbohF* was up-regulated in response to BL treatment. However, the transcript levels of *AtrbohD* and *AtrbohF* were lower upon ABA and BL co-treatment than with single hormone treatment (Fig. 4A). These gene expression patterns for ROS-producing enzymes correlated well with ROS production. Using a H_2_DCF-DA fluorescent dye, we examined ROS production in guard cells. We observed that upon ABA or BL treatment, ROS were produced specifically in the guard cells, and that ROS production was lower with the co-treatment of ABA and BL than with single hormone treatment, although the ROS level was still slightly above the basal level (Fig. 4B). Similar results were obtained in experiments investigating NO production. The expression of *NIA1* and *NIA2*, which encode two nitrate reductases in Arabidopsis (Desikan *et al.*, 2002), was also up-regulated following ABA or BL treatment. However, the expression of these genes decreased almost to basal levels under ABA and BL co-treatment (Fig. 5A). The patterns of NO production detected using DAF fluorescence were correlated with the patterns of *NIA1* and *NIA2* expression (Fig. 5B). These results indicate that BR negatively affects ABA-induced H_2_O_2_ and NO production, through the down-regulation of genes involved in ROS production.

**Fig. 6.**
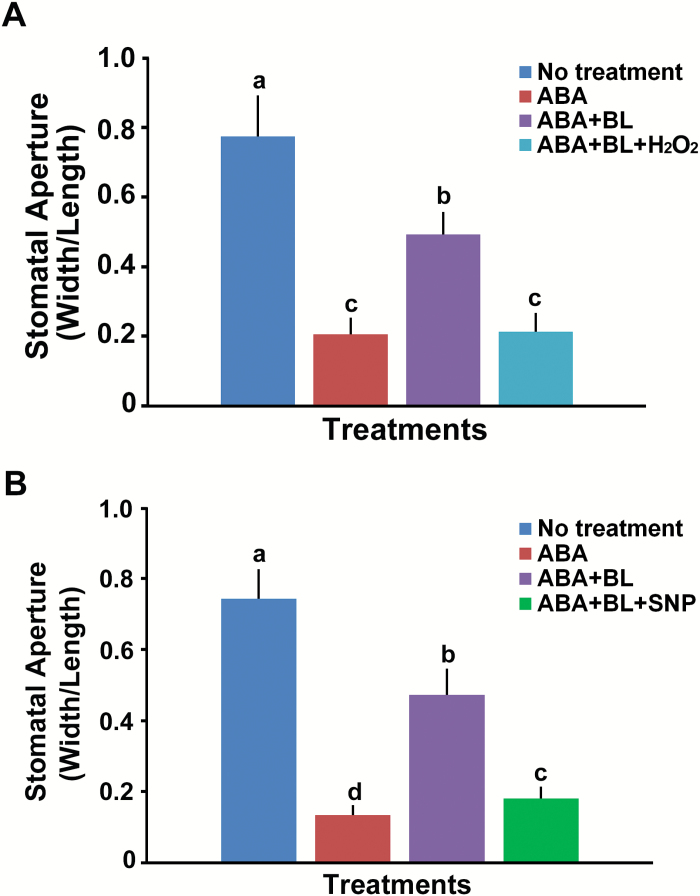
H_2_O_2_ or SNP treatment repressed the effect of BL on the inhibition of ABA-induced stomatal closure. (A) H_2_O_2_ (100 μM) or (B) SNP (100 μM) was applied 30 min after BL (1 μM) to the ABA-containing solution, and the cotyledons were incubated for a further 1 h before apertures were measured. Experiments were independently repeated twice (*n*=30 each time). Error bars indicate standard errors. Values labeled with different letters are statistically different analysed by one-way ANOVA (*P*<0.05). (This figure is available in color at *JXB* online.)

To test this hypothesis, we determined whether application of H_2_O_2_ and the NO donor sodium nitroprusside (SNP) could suppress the inhibiting effect of BR on ABA-induced stomatal closure. We sequentially added ABA, BL, and H_2_O_2_ or SNP to the solution at 30 min intervals and measured the stomatal aperture after 1 h of treatment with H_2_O_2_. We observed that 1 h treatment of H_2_O_2_ or SNP was sufficient to inhibit the effect of BR on stomatal opening in the presence of ABA (Fig. 6A, B).

### BR signaling capacity is required for ABA-induced stomatal closure

**Fig. 7.**
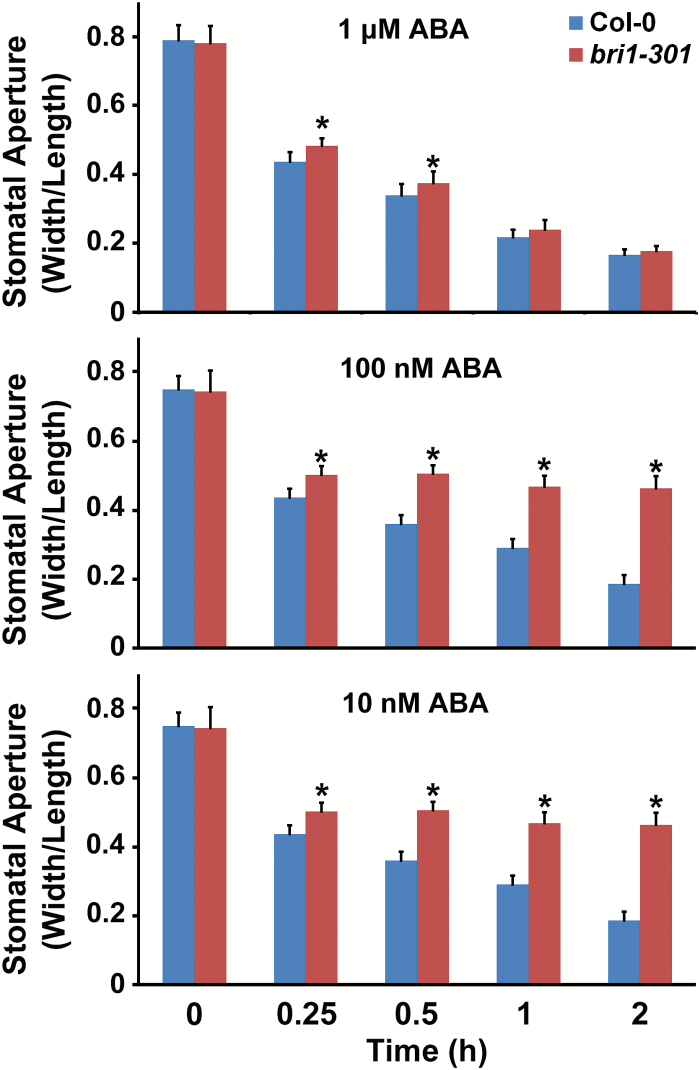
The *bri1-301* mutant showed reduced sensitivity to ABA for stomatal closure. Different concentrations of ABA (1 μM, 100 nM, and 10 nM) were added to the opening solution and the seedlings were incubated for the indicated times. Stomatal closure was measured as the width/length ratio of the cotyledon stomata. Experiments were independently repeated three times (*n*=25 each time). Error bars indicate standard errors (*P<0.0001, compared with the Col-0 plants under the same treatment, *t*-test). (This figure is available in color at *JXB* online.)

**Fig. 8.**
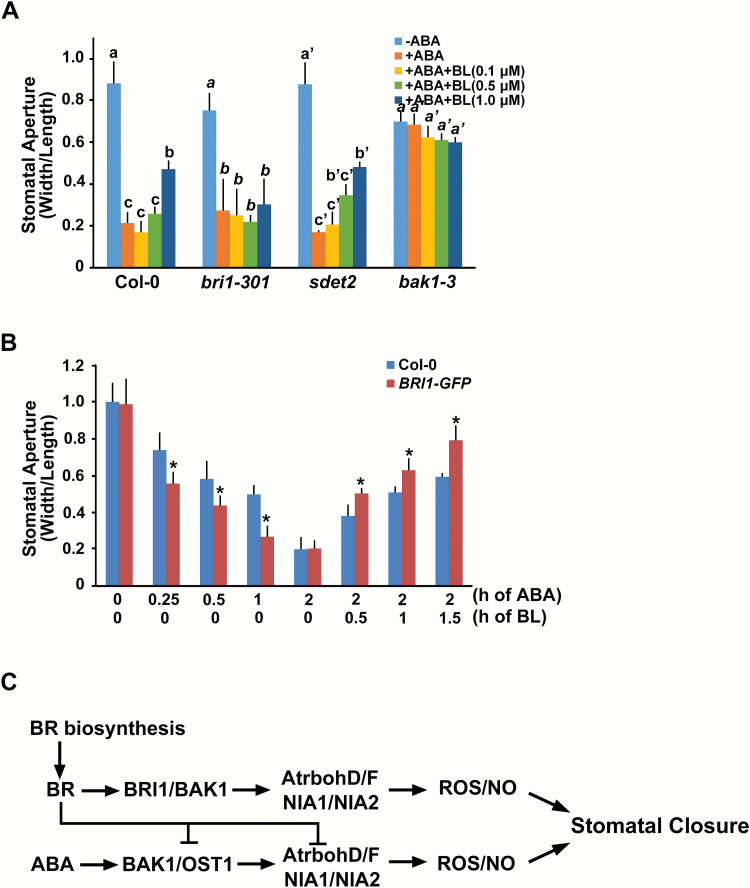
BRI1 is required for the BL inhibition of ABA-induced stomatal closure. (A) Comparison of the effect of BL on the inhibition of ABA-induced stomatal closure in various BR-related mutants. Indicated concentrations of BL were added to the 1 μM ABA-containing solution in which each mutant seedling was incubated. Stomatal closure was measured as a width/length ratio of the cotyledon stomata. Experiments were independently repeated three times (*n*=30 each time). Error bars indicate standard errors. Values labeled with different letters (roman, italic, primed letters, respectively) are statistically different analysed by one-way ANOVA (*P*<0.05). (B) ABA-induced stomatal closure of the BRI1-GFP plants was measured with or without BL treatment for the indicated times compared with wild type. Experiments were independently repeated three times (*n*=50 each time). Error bars indicate standard errors (*P<0.001, compared with the wild type plant under the same treatment). (C) Proposed model showing how BR induces stomatal closure and affects ABA-induced stomatal closure as well. (This figure is available in color at *JXB* online.)

To examine whether the inhibition of ABA-induced stomatal closure by BL occurs through the activation of BR signaling, we examined stomatal movement in the *bri1-301* mutant, which is a weak *bri1* mutant allele (Xu *et al.*, 2008). First, to confirm the ABA sensitivity of *bri1-301* mutant in stomatal closure, we measured the stomatal aperture of the *bri1-301* plants in response to various durations of ABA exposure with different ranges of ABA concentrations (Desikan *et al.*, 2002). Stomatal closing occurred later following short period of ABA treatment in the *bri1-301* mutant than in the wild type, although a 2 h treatment period was sufficient to close the stomata of *bri1-301* mutant to a similar degree to that observed in the wild type. Furthermore, decreased stomatal closure in the *bri1-301* mutant was more distinct upon treatment with lower concentrations of ABA (Fig. 7). These results indicate that ABA sensitivity was reduced in the *bri1-301* mutant. Upon treatment of BL alone, as expected, BL-induced stomatal closure was not observed in the *bri1-301* mutant and the *bak1-3* mutant (Supplementary Fig. S6). Based on these results, next, we monitored whether the inhibition of ABA-induced stomatal closure by BL was affected in the *bri1-301* mutant. We also confirmed the stomatal movements of the *bak1-3* and *sdet2* mutants in response to BL and ABA together for comparison. When 10-d-old cotyledons of each plant were incubated in an ABA-containing solution, the stomata of the *bri1-301* and *sdet2* mutants were closed within 2 h, consistent with the response in wild type plants. When plants were co-treated with BL and ABA, ABA-induced stomatal closure of the *sdet2* mutant was inhibited proportionally to the concentration of BL used, which was similar to the response observed in the wild type plant. However, ABA-induced stomatal closure of the *bri1-301* mutant was not inhibited by BL treatment (Fig. 8A). These results suggest that functional BRI1 is required for the regulation of stomatal movement: closure of stomata in response to BL and inhibition of ABA-induced stomatal closure. In comparison to the *bri1-301* mutant, the stomata of the *bak1-3* mutant were less sensitive to ABA (Fig. 8A), consistent with findings from a previous report (Shang *et al.*, 2016). BAK1 does not directly bind BL (Wang *et al.*, 2008), although it is important for BR signaling as a co-receptor of BRI1. Moreover, as BAK1 can function in guard cells by forming a complex with OST1 under ABA treatment (Shang *et al.*, 2016), ABA insensitivity in the *bak1-3* mutant may not be caused solely by the failure of BR signaling due to a lack of BAK1. When BL and ABA are both present, BAK1 can function as a versatile component in both signaling pathways. ROS production in the *bak1-3* mutant was not induced by ABA (Shang *et al.*, 2016). In this study, we also found that BL-induced ROS production did not occur in the *bak1-3* mutant, either, whereas ROS levels increased dramatically in the guard cells of wild type plants (Supplementary Fig. S7). Therefore, the stomatal aperture in the *bak1-3* mutant seemed to be the result of disturbances in both BR and ABA signaling pathways.

As reduced BR signaling led to the failure of BL to inhibit ABA-induced stomatal closure, we attempted to further validate the relationship between BR signaling capacity and stomatal movement using the *BRI1-GFP* transgenic plant, in which BR signaling capacity is increased by increasing the number of BRI1 receptors (Wang *et al.*, 2001; Kinoshita *et al.*, 2005). We observed that the *BRI1-GFP* plants were hyper-sensitive to ABA for stomatal closure when compared with wild type plants even in the absence of exogenous BL. Furthermore, the degree of inhibition of ABA-induced stomatal closure by BL was greater in *BRI1-GFP* transgenic plants than in wild type plants (Fig. 8B). Moreover, even in the presence of low concentration of BL, which did not affect stomatal movement in wild type plants, inhibition of ABA-induced stomatal closure was detected in the *BRI1-GFP* plants (Supplementary Fig. S8). These results show that *BRI1-GFP* transgenic plants were more sensitive than wild type plants to ABA and BL, such that they exhibited enhanced stomatal closure upon ABA treatment and enhanced inhibition of ABA-induced stomatal closure in the presence of BL. Therefore, BR signaling capacity affects ABA-induced stomatal closure positively and negatively through BRI1.

## Discussion

### BR alone promotes stomatal closure, but in concert with ABA, BR modulates ABA-induced stomatal closure both positively and negatively

Plants open stomata by regulating the turgor pressure of the two guard cells in order to obtain CO_2_ for photosynthesis, which results in over 95% of their water loss occurring through the stomatal pores (Buckley, 2005; Kim *et al.*, 2010). Therefore, plants have developed several multi-layered methods to regulate stomatal closure in an appropriate and timely manner for their survival (Carvalho *et al.*, 2015; Murata *et al.*, 2015; Scuffi *et al.*, 2016). We investigated how BR affects stomatal movement by itself and in combination with ABA. Although low concentrations of EBR (<100 nM) induced stomatal opening in tomato leaves, high concentrations (>1 μM) induced stomatal closure (Xia *et al.*, 2014), as in *Arabidopsis thaliana* (Shi *et al.*, 2015). In the present study, we describe the detailed kinetics of BL-induced stomatal closure in the presence of low and high concentrations of BL in wild type plants (Fig. 1B). Our result that even low concentration of BL induced stomatal closure differs from findings in tomato leaves treated with low concentrations of EBR (Xia *et al.*, 2014). However, it is difficult to directly compare relative effective doses of EBR and BL, and the possibility of different sensitivities to BRs in different tissues or different plant species cannot be ruled out.

In addition to the BR-induced stomatal closure, we also show that BRs can modulate ABA-induced stomatal closure both positively and negatively, depending on the BR concentration applied. Previous studies addressing the function of BRs in stomatal movements have examined stomatal responses of BR-biosynthetic, or signaling mutants in the presence of ABA (Ephritikhine *et al.*, 1999; Xue *et al.*, 2009). These studies showed that stomatal closure of the *det2* or *bri1-9* mutants was more sensitive to ABA, leading to the conclusion that BR functions negatively in stomatal closure. However, in the present study, we demonstrated that ABA sensitivity of the *bri1-301* mutant was partially defective when ABA was applied for shorter periods or when the concentration of ABA was low (Fig. 7), whereas ABA sensitivity of the *sdet2* mutant in the stomatal closure was not changed compared with wild type plant (Supplementary Fig. S3). When ABA was applied for longer periods or was followed by BL treatment (over 0.5 μM), ABA led to stomatal closure in the *sdet2* and *bri1-301* mutants to a similar degree as was observed in wild type plant (Figs 2A and 8A). However, inhibition of ABA-induced stomatal closure by BL occurred in the *sdet2* mutant, not in the *bri1-301* mutant (Fig. 8A), and the BR-biosynthetic inhibitor BRZ alleviated the BR repression on ABA-induced stomatal closure (Fig. 3C). These results strongly suggest that BRI1 needs to be functional in order to maintain ABA sensitivity and to transduce BR signaling to inhibit ABA-induced stomatal closure. These assumptions were strengthened by results in the *BRI1*-overexpressing transgenic plant *BRI1-GFP*, which displayed increased sensitivity to ABA in stomatal closure. Even low concentration of BL inhibited ABA-induced stomatal closure in the *BRI1-GFP* plants. Furthermore, BL inhibition of ABA-induced stomatal closure was enhanced in the *BRI1-GFP* plants (Fig. 8B). Therefore, these results suggest that under endogenous levels of BR, proper BRI1 function seems to be required for ABA-induced stomatal closure by maintaining ABA sensitivity. However, when the BR concentration exceeds a threshold level in the presence of ABA, BR inhibits ABA-induced stomatal closure by activating BR signaling. In this way, BR acts both positively and negatively to regulate stomatal movement, which is primarily governed by ABA.

### BL suppressed ABA-induced ROS and NO production, thereby inhibiting ABA-induced stomatal closure

ROS, including H_2_O_2_, and NO are important downstream signaling intermediates in various processes regulated by plant hormones (Simontacchi *et al.*, 2013; Sanz *et al.*, 2015; Xia *et al.*, 2015). Many studies have demonstrated that ABA (Pei *et al.*, 2000; Kwak *et al.*, 2003; Zhou *et al.*, 2014) and BR (Zhou *et al.*, 2014; Kang and Nam, 2016) induce ROS production. Usually during ABA-induced stomatal closure, OST1 promotes H_2_O_2_ production by activating ROS-producing enzymes (Kwak *et al.*, 2003). The resulting increase in H_2_O_2_ further induces an increase in the cytosolic Ca^2+^ level (Pei *et al.*, 2000). Therefore, our data showing that ROS production and the induction of genes involved in H_2_O_2_ and NO productions following treatment of ABA or BL alone are consistent with the findings of previous studies (Figs 4 and 5). When BR was applied to maize leaves, BR-induced NO production was observed, which subsequently activated ABA synthesis, resulting in enhanced water stress tolerance (Zhang *et al.*, 2011). In addition, we also found that ABA-induced H_2_O_2_ and NO production was repressed by co-treatment with BL (Figs 4 and 5). These observations were consistent with the observation that stomatal aperture was affected by ABA with or without BL treatment (Fig. 3), providing a possible explanation for why BL inhibited ABA-induced stomatal closure upon co-treatment. These results implied that, although ROS production can be induced in various ways, such as by ABA or BR treatment as shown in the present study, it is not cumulative. Simultaneous ROS production by various stimuli should be regulated in order to maintain homeostasis to prevent the excessive accumulation of ROS, which may be harmful for cellular processes. For example, ethylene played a positive role in ROS production but also induced synthesis of flavonols, which function as antioxidants, leading to slower stomatal closure (Watkins *et al.*, 2014). Xia *et al.* detected dynamic changes of ROS production depending on the concentrations of EBR (Xia *et al.*, 2014). Transient increases in ROS production induced by low concentrations of EBR are required for EBR-induced stomatal opening in tomato leaves. However, prolonged ROS production was observed in guard cells upon application of high concentrations of EBR, resulting in stomatal closure. These results suggest that high levels of ROS function downstream of ABA and BL signaling in the process of stomatal closure. The requirement of ROS and NO production for stomatal closure was confirmed by direct treatment of H_2_O_2_ or SNP in the ABA- and BL-containing solution. Both H_2_O_2_ and SNP suppressed the inhibitory effect of BL on ABA-induced stomatal closure (Fig. 6A, B). Taken together, these results indicate that BRs negatively affect ABA-induced H_2_O_2_ and NO production, through the down-regulation of genes involved in ROS production.

### Interactions of ABA and BR signaling are important for the regulation of stomatal closure

Based on these and our previous results showing that BAK1 acts in ABA signaling through the interaction with OST1 in the regulation of stomatal closure triggered by ABA (Shang *et al.*, 2016), we propose that BR inhibition of ABA-induced stomatal closure occurs in two ways. One is through the activation of BR signaling. As discussed, application of BR to stomata closed in response to ABA did not affect stomatal movement in the *bri1-301* mutant (Fig. 8A). However, application of BR to stomata closed in response to ABA caused the stomata to open to a greater degree in the *BRI1-GFP* plants than in wild type plants (Fig. 8B). In addition, even when ABA synthesis is disturbed, closed stomata were normally observed in *aba2-1* in response to BL, as the BR-signaling capacity in this mutant is intact (Fig. 2B). A second way is through BAK1, which was originally identified as a co-receptor of BRI1 (Li *et al.*, 2002; Nam and Li, 2002). BAK1 was subsequently shown to be a co-receptor of FLS2, which is a flagellin-binding receptor involved in plant immunity (Chinchilla *et al.*, 2007; Sun *et al.*, 2013; Koller and Bent, 2014), and of ER and ERL1, which are EPF2 and EPF1 receptors for stomatal development and patterning (Meng *et al.*, 2015). These results suggest that BAK1 can act in multiple pathways to regulate plant development. Here, we demonstrated that the *bak1-3* mutant also showed defects in BL-induced stomatal closure and BL-induced ROS production (Supplementary Figs S6 and S7). We previously showed that ABA-induced stomatal closure was abolished in the *bak1* mutant. Therefore, as shown in Fig. 8A, in the presence of both BR and ABA, the stomatal movement of the *bak1* mutant was prevented. ABA-induced *OST1* expression and ROS production were also impaired in the *bak1* mutant. These results suggest that BAK1 has a dual function in ABA and BR signaling. Therefore, in addition to the inhibition of ABA-induced ROS and NO productions by BL, it is possible that BL can affect ABA-induced stomatal closure by inhibiting the interaction between BAK1 and OST1 (Shang *et al.*, 2016), which lead to recruitment of further BAK1 to BRI1, leading to stomatal reopening (Fig. 8C). Taken together, these results suggest that interactions between ABA and BR signaling are important for fine-tuning the regulation of stomatal closure throughout the plant lifespan.

## Supplementary data

Supplemental data are available at *JXB* online.

Figure S1. Brassinolide (BL) induced stomatal closure.

Figure S2. Phenotypes of the *sdet2* mutants.

Figure S3. Stomatal aperture in *sdet2* mutant in response to various concentration of ABA.

Figure S4. Stomatal aperture in *dwf4-1*, *aao3-4* and *abi1-1* mutants in response to ABA or BL.

Figure S5. ABA-induced stomatal closure was inhibited by BL even in the presence of high concentration of ABA.

Figure S6. BL-induced stomatal closure was not observed in *bri1-301* and *bak1-3* mutants.

Figure S7. ROS productions in *bak1-3*.

Figure S8. *BRI1-GFP* plants showed higher sensitivity to BL in the inhibition of ABA-induced stomatal closure.

Table S1. List of primers used in this study.

Supplementary Data
