# Supplementary material for: Brassinosteroids modulate ABA-induced stomatal closure in Arabidopsis
Source: J Exp Bot. 2016 Oct 17;67(22):6297–308. doi: 10.1093/jxb/erw385 (PMC5181576; doi:10.1093/jxb/erw385)
Supplement: Supplementary Data [file supp_67_22_6297__index.html]

Brassinosteroids modulate ABA-induced stomatal closure in Arabidopsis — Brassinosteroids modulate ABA-induced stomatal closure in Arabidopsis — Supplementary Data 

# Brassinosteroids modulate ABA-induced stomatal closure in Arabidopsis

## Supplementary Data

Data files

- supplementary\_table\_S1\_figures\_S1\_S8.pdf - Supplementary Data
