# Supplementary material for: Brassinosteroids modulate ABA-induced stomatal closure in Arabidopsis
Source: J Exp Bot. 2016 Oct 17;67(22):6297–308. doi: 10.1093/jxb/erw385 (PMC5181576; doi:10.1093/jxb/erw385)

Supplemental Table 1. Primer sequences used in this study

Primes Name	Sequence
qAtrbohD F	5'-GGTGCCCATTTAACGTGTGATT-3'
qAtrbohD R	5'-CGGCTCATAGGTGTCCTCATC-3'
qAtrbohF F	5'-CGAGATCTCCGACGATGAAAC-3'
qAtrbohF R	5'-AGCGGCGGTACTAGTAGTGG-3'
qNIA1 F	5'-GAAAATCCCGGTTAGGCTCAT-3''
qNIA1 R	5'-AATGCGAATCGGAACTTACGA-3'
qNIA2 F	5'-CCGTTGATGTGGTTGGCTACT-3'
qNIA2 R	5'-TTAGGGAATCTTGGGTGGACA-3'
qUBQ5 F	5'-CGGACAGCAGCGATTG-3'
qUBQ5 R	5'-GGGTACGGCCGTCTTCAAG-3'

Supplemental Figure Legends

Supplemental Figure S1. Brassinolide (BL) induced stomatal closure. Various concentration of BL indicated was added to the opening solution and the seedlings were incubated for 15 min or 2 hours. Stomatal closure was measured as the width/length ratio of the cotyledon stomata. Experiments were independently repeated three times (n = 35 each time). Error bars indicate standard errors. Values labeled with different letters (regular, italic, primed letters respectively) are statistically different analyzed by one-way ANOVA (P < 0.05).

Supplemental Figure S2. Phenotypes of the *sdet2* mutants. (A) Gross morphology of *sdet2* plants compared with that of wild type plants, Col-0. Pictures were taken of plants grown for 4 weeks under long-day conditions. (B) *Sdet2* plants can sense BR. BL (1 μM) was applied to the 7-d seedlings grown on 1/2 MS media. Pictures were taken after 24 h.

Supplemental Figure S3. Stomatal aperture in *sdet2* mutant in response to various concentration of ABA. ABA was added to the opening solution and the seedlings were incubated for an additional 2 h. The stomatal aperture was determined as the width/length ratio of the cotyledon stomata. Experiments were independently repeated twice (n = 25 each time). Error bars indicate standard errors. Values labeled with different letters (regular and italic, respectively) are statistically different analyzed by one-way ANOVA (P < 0.05).

Supplemental Figure S4. Stomatal aperture in (A) *dwf4-1*, (B) *aao3-4* and (C) *abi1-1* mutants in response to ABA or BL. ABA (1 μM) or BL (1 μM) was added to the opening solution and the seedlings were incubated for an additional 2 h. The stomatal aperture was calculated as the width/length ratio of the cotyledon stomata. Experiments were independently repeated twice (n = 25 each time for *dwf4-1* and *aao3-4*, n = 30 each time for *abi1-1*). Error bars indicate standard errors. In (A) and (B), *: P<0.0001, compared to the corresponding samples without treatment by t-test. In (C), Values labeled with different letters are statistically different analyzed by two-way ANOVA (P < 0.05).

Supplemental Figure S5. ABA-induced stomatal closure was inhibited by BL even in the presence of high concentration of ABA (10 μM). ABA-induced stomatal closure was measured with or without BL treatment for the indicated times. Experiments were independently repeated twice (n = 50 each time). Error bars indicate standard errors. (*: $P<0.0001$, compared to the samples without ABA treatment, **: $P<0.0001$ compared to the samples treated with ABA only, t-test).

Supplemental Figure S6. BL-induced stomatal closure was not observed in *bri1-301* and *bak1-3* mutants. BL (1 μM) was added to the opening solution and the seedlings were incubated for an additional 2 h. The stomatal aperture was determined as the width/length ratio of the cotyledon stomata. Experiments were independently repeated twice (n = 30 each time). Error bars indicate standard errors. Values labeled with different letters are statistically different analyzed by two-way ANOVA ($P < 0.05$).

Supplemental Figure S7. ROS productions in *bak1-3*. ROS production in *bak1-3* compared to wild type in response to BL was detected by fluorescent H_2DCF-DA in the guard cells. Size bar indicates 20 μm.

Supplemental Figure S8. *BRI1-GFP* plants showed higher sensitivity to BL in the inhibition of ABA-induced stomatal closure. ABA-induced stomatal closure in the *BRI1-GFP* plants was measured and compared with that in the wild type plants in the presence or absence of BL. Low concentration BL (10 nM) was added to the opening solution and the seedlings were incubated for an additional 2 h. Experiments were independently repeated three times (n = 50 each time). Error bars indicate standard errors. (*: $P<0.001$, compared to the wild type samples under the same treatment, t-test)

Ha *et al.*, Supplemental Figure S1

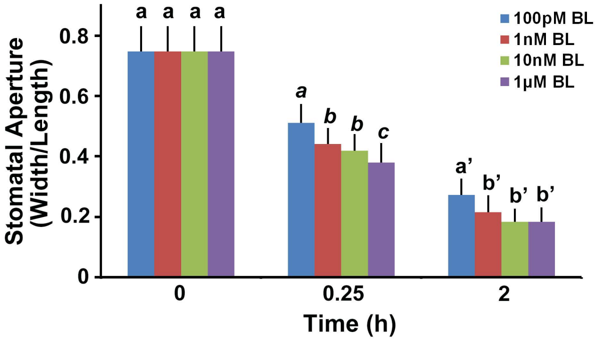

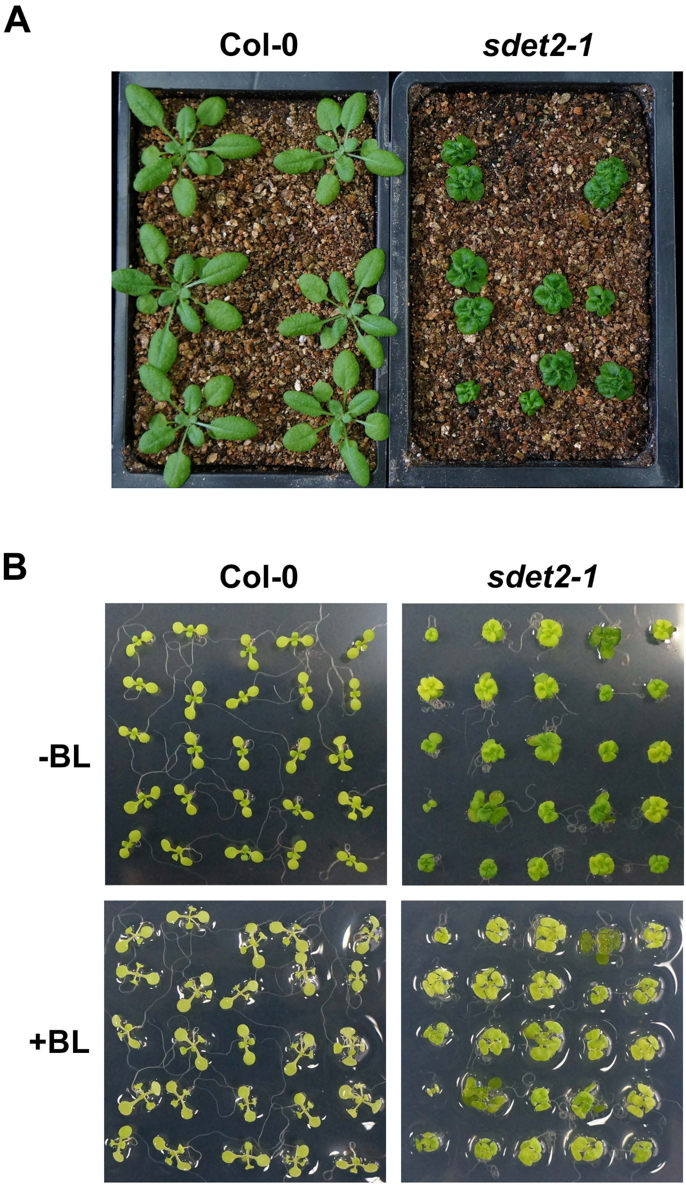
A
Col-0
sdet2-1
B
Col-0
sdet2-1
-BL
+BL

Ha *et al.*, Supplemental Figure S3

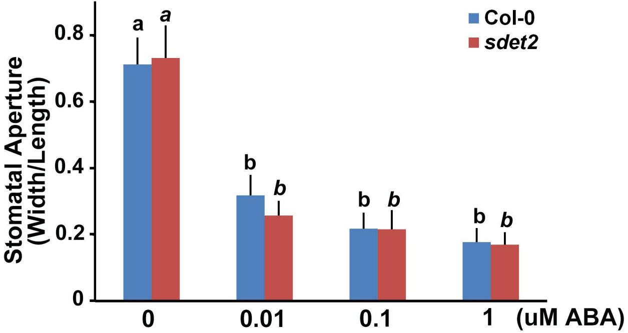

Ha *et al.*, Supplemental Figure S4

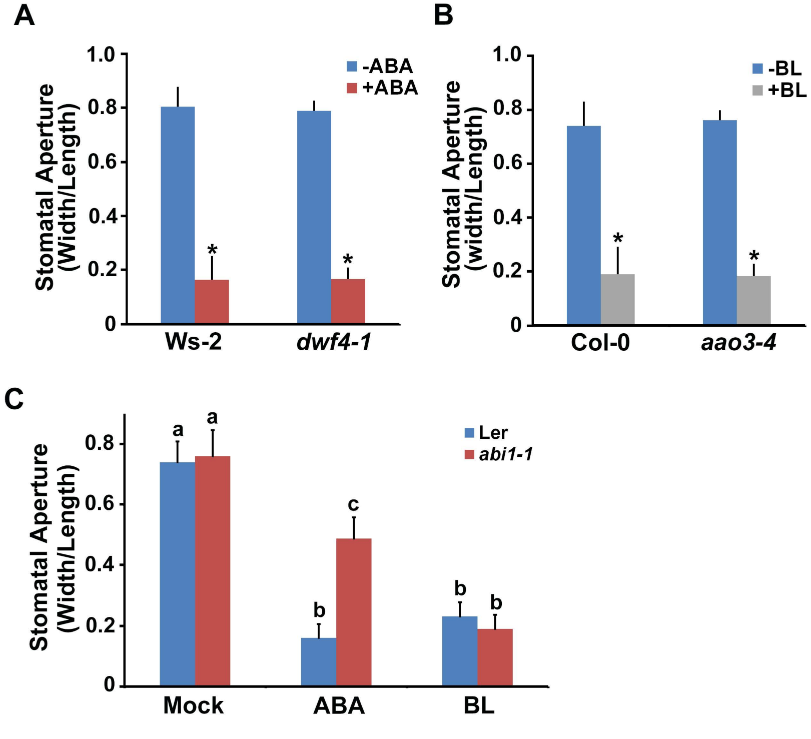

Ha *et al.*, Supplemental Figure S5

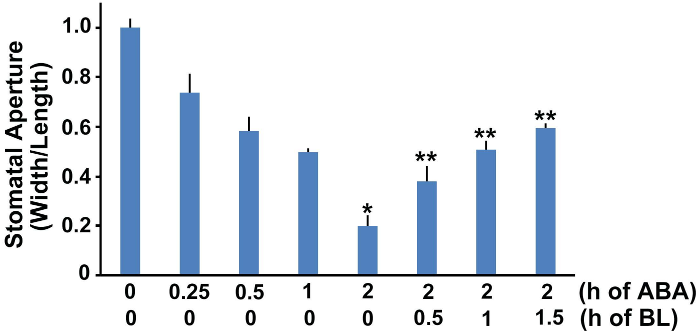

Ha *et al.*, Supplemental Figure S6

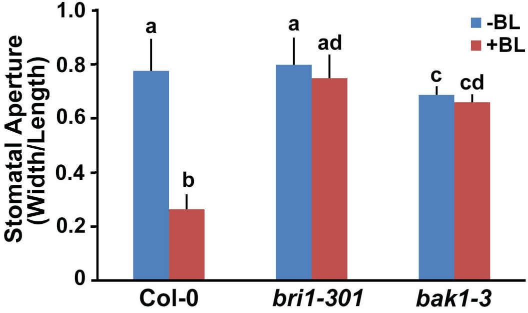

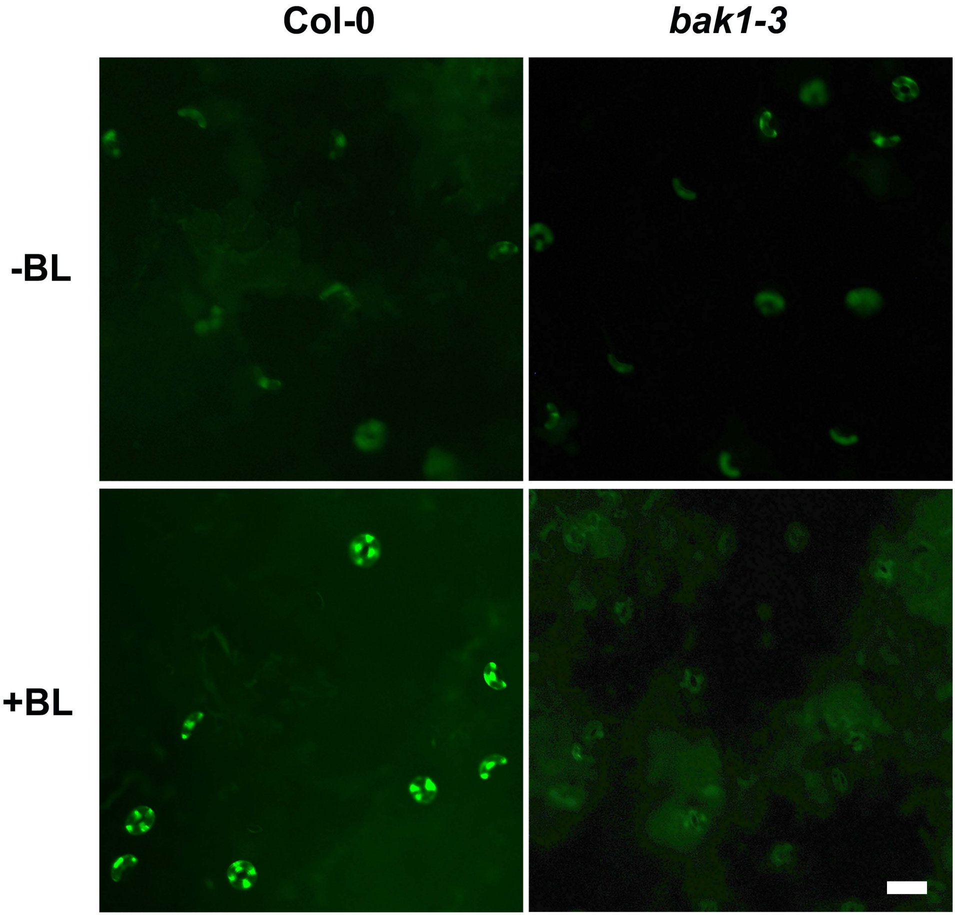
Col-0
bak1-3
-BL
+BL

Ha *et al.*, Supplemental Figure S8

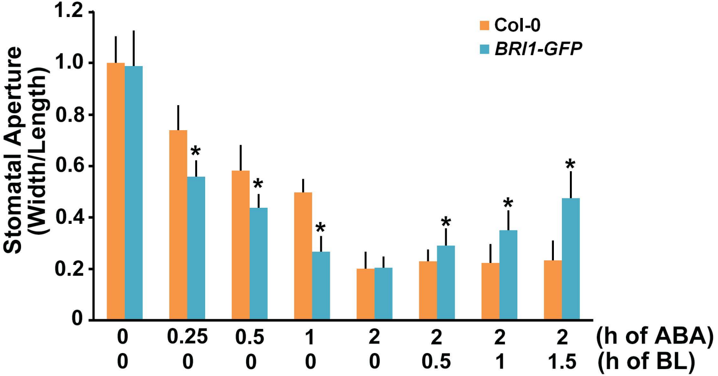

Supplement: Supplementary Data [file supp_erw385_supplementary_table_S1_figures_S1_S8.pdf]
